# Evaluating the agronomic efficiency of alternative phosphorus sources applied in Brazilian tropical soils

**DOI:** 10.1038/s41598-024-58911-0

**Published:** 2024-04-12

**Authors:** Lucas Jónatan Rodrigues da Silva, Aline da Silva Sandim, Ana Paula Rodrigues da Silva, Angélica Cristina Fernandes Deus, João Arthur Antonangelo, Leonardo Theodoro Büll

**Affiliations:** 1 Department of Forest Science, Soils and Environment, College of Agronomic Sciences, São Paulo State University, Botucatu, SP 18610-307 Brazil; 2 Department of Plant Protection, Rural Engineering and Soils, College of Engineering, São Paulo State University, Ilha Solteira, SP 15385-000 Brazil; 3 Department of Crop and Soil Sciences, Washington State University, Pullman, WA USA

**Keywords:** Phosphorus fertilizers, Nutrient accumulation, Phosphorus use efficiency, Environmental sciences, Element cycles, Plant sciences

## Abstract

Understanding the efficacy of alternative phosphorus (P) sources in tropical soils is crucial for sustainable farming, addressing resource constraints, mitigating environmental impact, improving crop productivity, and optimizing soil-specific solutions. While the topic holds great importance, current literature falls short in providing thorough, region-specific studies on the effectiveness of alternative P sources in Brazilian tropical soils for maize cultivation. Our aim was to assess the agronomic efficiency of alternative P sources concerning maize crop (*Zea mays* L.) attributes, including height, shoot dry weight, stem diameter, and nutrient accumulation, across five Brazilian tropical soils. In greenhouse conditions, we carried out a randomized complete block design, investigating two factors (soil type and P sources), evaluating five tropical soils with varying clay contents and three alternative sources of P, as well as a commercial source and a control group. We evaluated maize crop attributes such as height, dry weight biomass, and nutrient accumulation, P availability and agronomic efficiency. Our results showed that, although triple superphosphate (TSP) exhibited greater values than alternative P sources (precipitated phosphorus 1, precipitated phosphorus 2 and reactive phosphate) for maize crop attributes (e.g., height, stem diameter, shoot dry weight and phosphorus, nitrogen, sulfur, calcium and magnesium accumulation). For instance, PP1 source increased nutrient accumulation for phosphorus (P), nitrogen (N), and sulfur (S) by 37.05% and 75.98% (P), 34.39% and 72.07% (N), and 41.94% and 72.69% (S) in comparison to PP2 and RP, respectively. Additionally, PP1 substantially increased P availability in soils with high clay contents 15 days after planting (DAP), showing increases of 61.90%, 99.04%, and 38.09% greater than PP2, RP, and TSP. For Ca and Mg accumulation, the highest values were found in the _C_Oxisol_2_ soil when PP2 was applied, Ca = 44.31% and 69.48%; and Mg = 46.23 and 75.79%, greater than PP1 and RP, respectively. Finally, the highest values for relative agronomic efficiency were observed in _C_Oxisol_2_ when PP1 was applied. The precipitated phosphate sources (PP1 and PP2) exhibited a similar behavior to that of the commercial source (TSP), suggesting their potential use to reduce reliance on TSP fertilization, especially in soils with low clay contents. This study emphasized strategies for soil P management, aimed at assisting farmers in enhancing maize crop productivity while simultaneously addressing the effectiveness of alternative P sources of reduced costs.

## Introduction

Maize (*Zea mays* L.) is one of the most important crops worldwide, used as energy source for both humans and animals, and as a feedstock for biofuel production^1^. According to data from the Companhia Nacional de Abastecimento^2^, Brazil's maize production reached approximately 131.9 million tons in the 2022/2023 agricultural season. This high maize production can be attributed to improved fertilizer management, including the use of phosphorus (P) fertilizers^3^. Phosphorus has a crucial role on crops growth and yield, participating in physiologic process (e.g., photosynthesis), plant metabolism, biosynthesis of organic compounds, nucleic acids and phospholipids, enzymatic activities, gene regulation, and signaling^4^.

The low phosphorus (P) efficiency in Brazilian cropland is attributed to soil chemical characteristics, such as the presence of variable charges, low soil pH, and high P sorption capacity resulting from the presence of iron and aluminum sesquioxide’s. Therefore, the correct management must be considered to increase crop yields under these conditions^5^. Also, the conventional P sources, due to their high solubility, remain in the soil solution for only a short period of time before rapidly undergoing chemisorption^6^.

According to Volf and Rosolem^7^, excessive amounts of conventional P fertilizers are often applied across extensive areas to overcome the soil sorption capacity, resulting in expensive management costs in some cases. Annually, it is estimated that 15 million tons of P sources are deposited in the croplands, and each year 12 million tons are lost^1^. In Brazilian tropical soils, it is estimated that P removal by crops ranges from 30 to 60% of the total applied; in four years this value could be 52% of total P applied, while over 70% remain accumulated in the soils^8^.

It is estimated that 80% of all fertilizer consumed in Brazilian crop nutrition is imported from other countries every year. This international dependence has led to an increase in fertilizer costs due to conflicts in Eastern Europe^9^. Alternative fertilizers generated through industrial processes are considered potential products in agriculture. The manufacturing process of triple superphosphate (TSP) consists in the use of H_3_PO_4_ (phosphoric acid) to treat the surface of phosphate rock. During the treatment of phosphate rocks with phosphoric acid, some products are generated (e.g., phosphogypsum and precipitated compounds), usually these products are stored in stabilization ponds^10^. This can represent an environmental problem and costs for the fertilizers industry. On the other hand, these residues could be used in agriculture, reducing the environmental problems and promoting P for crops.

Some studies had demonstrated the positive effects of industrial by-products in crops^11–13^. Studies done by Leal et al.^10^, described that TSP manufacturing residues had a positive effect under grasses development, mainly after successive cuts, indicating their residual effect under acid pH condition in sandy soil. These results are supported by Sandim et al.^14^, who showed the high levels of moderately and labile P forms, indicating the gradual P release in tropical soils. On the other hand, remains uncertain the agronomic efficiency of these products in Brazilian tropical soils with varied clay contents. Our aim was to evaluate the agronomic efficiency of three alternative P sources compared to TSP in terms of maize crop attributes, including height, shoot dry weight, stem diameter, and nutrient accumulation, across five Brazilian tropical soils. Building upon the study conducted by Sandim et al. ^14^, we expected to observe an increase in nutrient accumulation and biomass weight in maize plants due to adequate P management, even when cultivated in soils with varying clay contents. We hypothesize that alternative P sources can enhance P availability for crops and potentially reduce reliance on TSP fertilization.

## Material and methods

The experiment was devised to compare a conventional, highly water-soluble phosphate fertilizer (TSP), against two distinct P sources derived from the phosphate fertilizer manufacturing process, alongside a sedimentary phosphate rock. These materials were applied in five soil types, varying P levels and clay contents (Sandy loam Entisol; Loamy sand Entisol; Clayey Oxisol 1; Sandy clay loam Oxisol; Clayey Oxisol 2)^15^.

### Soil chemical attributes

Soil samples were collected from two farms, Lageado (Botucatu) and São Manoel, located in the state of São Paulo, Brazil. The geographical coordinates of these farms are approximately 22° 50′19″ S, 48°25′54″ W, at an elevation of 738 m.a.s.l, and 22°44′28′′ S, 48°34′37′′ W, at an elevation of 740 m.a.s.l, respectively. Before the implementation of the experiment, the studied five soils were submitted to chemical and particle size analysis. The soil chemical and particle size attributes are presented in Table 1.

**Table 1:** Chemical and physical soil properties of the five soils varying clay contents before to start the greenhouse experiment.

Soils	pH	SOM	P	H + Al	K	Ca	Mg	SB	CEC	BS	PMAC	Sandy	Clay	Silt
	CaCl_2_	g dm^−3^	mg dm^−3^	mmol_c_ dm^−3^						%	mg kg^−1^	g kg^−1^		
_SL_Entisol	4.1	7.0	2.0	29.0	1.2	5.0	1.0	8.0	36.0	21.0	243.43	802	135	65
_LS_Entisol	4.5	10.0	2.0	23.0	2.0	9.0	5.0	15.0	38.0	40.0	113.51	866	84	50
_C_Oxisol_1_	4.2	23.0	8.0	76.0	1.9	5.0	3.0	10.0	86.0	12.0	1055.4	163	584	253
_SCL_Oxisol	3.8	32.0	3.0	94.0	1.4	2.0	1.0	5.0	99.0	5.0	388.14	669	274	57
_C_Oxisol_2_	4.0	33.0	5.0	76.0	2.1	7.0	3.0	12.0	89.0	14.0	705.47	360	510	130

*SB* sum of bases, *CEC* cation exchange capacity, *BS * base saturation, *PMAC*  phosphorus maximum adsorption capacity.

_*SL*_*Entisol* sandy loam Entisol, _LS_*Entisol* loamy sand *Entisol*, _C_*Oxisol*_1_ clayey Oxisol, _SCL_*Oxisol* sandy clay loam Oxisol, _C_*Oxisol*_2_ clayey Oxisol.

For initial screening of soil chemical and particle size attributes, samples were collected at a depth of 0.20 m. Soil attributes, including pH, soil organic matter (OM) content, available P, K^+^, Ca^2+^, Mg^2+^, sum of bases (SB), cation exchange capacity (CEC), and base saturation (BS%), were determined. Soil pH was measured using a CaCl_2_ 0.01 mol L^−1^ solution^16^. Soil OM was determined using the rapid dichromatic oxidation method^17^. Soil exchangeable cations (available P, K^+^, Ca^2+^, and Mg^2+^) were extracted using ion-exchange using ion exchange resin in a soil:resin:water (1:1:10, v/v). The samples were shaken for 16 h on an orbital shaker at 220 oscillations min^–1^. The P concentration of the extract was performed by phosphomolybdenum blue complex formation in sulfuric medium and with ascorbic acid as reducing agent, using a spectrophotometer. The exchangeable Ca, Mg, and K concentrations were determined by absorption spectrophotometry^16^. Also, the potential acidity (H + Al) was determined using calcium acetate ^16^. The SB, cation exchange capacity (CEC), and base saturation (BS%) were determined following Raij et al. (2001). For the particle size analysis was determined by Claessen^18^.

The P maximum adsorption capacity of the five studied soils was determined by shaking soil samples in a 0.01 mol L^−1^ CaCl_2_ solution at a ratio of 1:10 (soil: solution) for 24 h, with varying concentrations of P ranging from 0 to 200 mg L^−1^. The samples were then centrifuged at 3000 rpm for 15 min, and the remaining P in the solution was quantified using molybdate blue and ascorbic acid ^19^. Finally, the P maximum adsorption capacities were determined using Langmuir's Isotherms^20^.

### Chemical assessment of P fertilizers

To evaluate the agronomic efficiency of the examined phosphorus (P) fertilizers, we opted for three alternative P sources. Among these, two were derived from distinct stages of triple superphosphate (TSP) manufacturing (PP1 and PP2). PP1 originated from the production process of phosphoric acid, involving the precipitation reaction of dilute H_3_PO_4_ with lime or calcium hydroxide. This process led to sedimentation, forming a dried powder^10^. On the other hand, PP2 was obtained through a physico-chemical reaction involving phosphate, iron, and aluminum salts. This reaction resulted in the formation of positively charged metallic ions, which subsequently reacted with negatively charged phosphate ions. The outcome was the creation of fine flakes that underwent precipitation during a flocculation phase; the products are manufactured in Minas Gerais, Brazil. The third source is reactive phosphate (RP) from Sechura desert region of Peru. This source derived from the decomposition of marine residues. These alternatives were then compared with a reference P source, triple superphosphate‒TSP. The chemical and physical properties of the P sources were characterized in accordance with MAPA^21^ (Table 2).

**Table 2:** Chemical characterization of P sources used in the greenhouse experiment.

Sources	P_2_0_5_-T	P_2_O_5_-CA	P_2_O_5_ NAC	WP_2_O_5_	K_2_O	SO_4_	Ca	Mg	Cu	Fe	Mn	Zn	B
PP1	9.10	2.62	3.53	–	–	3.60	24.76	0.23	–	1.05	0.3	0.05	0.08
PP2	14.40	5.97	11.68	–	–	2.50	15.0	0.34	0.01	3.29	0.8	0.08	0.07
RP	29.40	6.87	5.77	–	0.04	32.60	32.50	0.33	0.02	0.39	0	0.06	0.09
TSP	48.08	17.04	45.07	37.34	–	4.40	13.10	0.26	0.02	1.79	0.07	0.08	0.02

*TP*_2_*O*_5_  total phosphate, *P*_2_*O*_5_ *CA*  citric acid, *P*_2_*O*_5_* NAC* neutral ammonium citrate,* W*  water. Methods applied were performed according to the MAPA (2017).

*PP1* precipitated P,* PP2* precipitated P,* RP* reactive phosphate rock,* TSP* triple superphosphate.

### Experimental design

**Figure 1:**
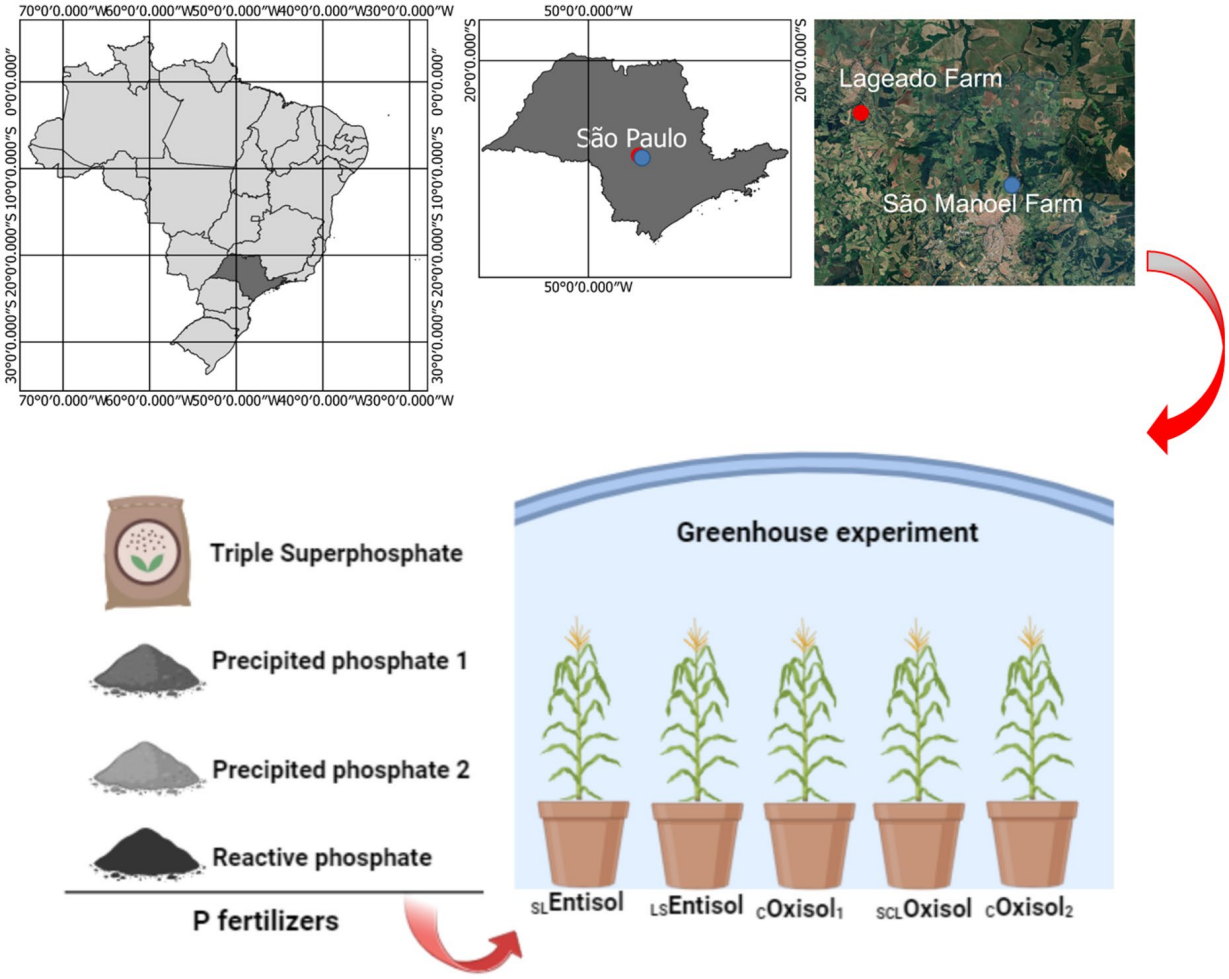
Location and experimental design of each studied P source and different soil types, Botucatu, São Paulo, Brazil. _*SL*_*Entisol* sandy loam Entisol, _*LS*_*Entisol* loamy sand Entisol, _*C*_*Oxisol*_*1*_ clayey Oxisol, _SCL_*Oxisol*  sandy clay loam Oxisol, _C_*Oxisol*_2_ clayey Oxisol.

In a greenhouse experiment, we analyzed the effects of three alternative P sources on the agronomic attributes of *Zea mays* L., cultivated at five soil types. In greenhouse conditions, we carried out a randomized complete block design, investigating two factors (soil type content and P sources), evaluating five tropical soils with varying clay contents and three alternative sources of P, as well as a commercial source and a control group with four replicates (*n* = 4). Each plastic pot had a volume of 19 dm^3^, representing one experimental unit, totaling one hundred experimental units (Fig. 1).

### Greenhouse experiment

Before the experiments began, the soils were sieved through a 4 mm mesh to remove roots, straw and clods and were then placed in the pots. The initial soil pH was adjusted by BS% method to meet the requirements of the maize crop (BS% = 70%) using dolomitic limestone (Relative Neutralizing Value = 98%). The soils remained properly moistened (70% of the field capacity‒FC) to allow for limestone reaction, according to Raij et al.^22^. After 30 days of limestone application, five maize plants were cultivated in plastic pots, remaining two plants per pot, each containing 19 dm^−3^ of soil. Each experimental unit was fertilized with 120 mg P dm^−3^ (furrow application), 120 mg K dm^−3^ and 60 mg N dm^−3^. The nitrogen (N) and potassium (K) fertilization were divided into two applications: at 15 and 30 DAP emergence. Additionally, micronutrients (B, Zn, Cu, Fe, and Mn) were applied via soil in silicate oxide form in each plot as recommended by Raij et al.^22^. All fertilizers were applied based on initial nutrient content on soil and meeting crop requirements for 10–12 t ha^−1^ yield goal. The pots were watered daily and maintained at 70% of FC, with water levels monitored by weighing.

### Plant morphological attributes and nutrient accumulation

Plant measurements were taken on 55 DAP emergence, including: (i) stem diameter (at 1 cm above the soil; (ii) plant height; (iii) shoot dry weight; (iv) accumulated nutrients in shoot. The shoot dry weight (in grams) was determined by drying the samples for 48 h at 65 °C. To determine the nutrients accumulated in the shoot dry weight, the samples were ground in a knife mill and passed through a 2-mm sieve. To determine the nutrients content in the shoot dry weight, we used the methodology described by Malavolta et al.^23^. The nutrient accumulation was calculated as follows: Nutrient accumulation (g kg^−1^) = dry weight (g plant^‒1^) × nutrient content (g kg^−1^) ^24^.

### Soluble phosphorus

The soluble P content was determined twice during the greenhouse experiment (15 and 30 DAP emergence) and followed the porous capsules extractor method^25^. Initially, ceramic porous capsules (6 cm height × 19 mm diameter) were installed at a depth of 10 cm. Then, a tension of 70 kPa was applied through a vacuum pump, and the extracted soil solution was packed in the plastic pots. After, the P contents in the soil solution was determined by colorimetry according to Raij et al. ^16^.

### Relative agronomic efficiency

Relative agronomic efficiency (RAE) was calculated by dividing each P source treatment to the reference fertilizer (TSP) added at the same fertilization dose, following the equation proposed by Büll et al.^26^:

$$RAE \left(\%\right)=\frac{YaP-Control}{YrP-control} \times 100$$

where RAE (%) is the relative agronomic efficiency, *YaP* is dry matter production by plants in a given alternative P fertilizer (mg plant^−1^); *YrP* is the dry matter production by plants in the reference P fertilizer (TSP).

### Statistical analysis

Prior to data analysis, all dataset was tested for normality with Shapiro–Wilk’s test (“shapiro.test” function), next all variables were analyzed with a two-way ANOVA with the main factor the P source used, the soil type as secondary factor, and pots number as a random factor. Tukey’s test was used as the post hoc test (*p* < 0.05). We performed principal component analysis (PCA) to outline the relationship between soil type and plant nutrient contents. All statistical analyses were performed using the packages *Hmisc, psych, car, ade4,* and *vegan* using R core Team^27^.

### Experimental research and field studies on plants including the collection of plant material

The authors declare that the cultivation of plants and carrying out study in São Paulo State University (UNESP), complies with all relevant institutional, national and international guidelines and treaties.

## Results

### Plant height, stem diameter and shoot dry weight

The plant height, stem diameter, and shoot dry weight were affected differently depending on the fertilizer (Table 3). The TSP source showed the highest values (e.g., plant height, stem diameter, and shoot dry biomass) in all studied soils. On the other hand, among alternative P sources, PP1 showed to be the most efficient one, increasing the plant height of up to 15.87% and 54.96% in comparison to PP2 and RP sources in the _C_Oxisol_2_, respectively. The PP2 source increased stem diameter by up to 14.68% and 47.25% higher than PP1 and PR, respectively. The PP1 source increased the shoot dry weight of up to 41.24% and 73.41% when compared to PP1 and PR.

**Table 3:** Plant height, stem diameter and shoot dry weight (mean ± SD) as a function of alternative P sources application in five tropical soils with different clay contents.

Soils	P Sources				
	Control	PP1	PP2	RP	TSP
Plant height (cm)					
_SL_Entisol	34.37 ± 6.42 Bd	123.12 ± 12.54 Ab	81.25 ± 14.18 Bc	50.12 ± 9.49 Bc	140.87 ± 4.15 Aa
_LS_Entisol	42.25 ± 4.66 Bd	119.25 ± 10.29 Ab	112.76 ± 7.73 Ab	58.25 ± 2.84 Bc	134.87 ± 4.60 Aa
_C_Oxisol_1_	39.37 ± 8.17 Bd	105.33 ± 14.29 Bb	93.04 ± 4.11 Bb	54.62 ± 5.72 Bc	128.50 ± 10.09 Aa
_SCL_Oxisol	43.00 ± 10.20 Bd	106.87 ± 14.76 Bb	75.87 ± 7.76 Bc	37.12 ± 6.03 Cd	142.87 ± 9.06 Aa
_C_Oxisol_2_	71.62 ± 13.64 Ac	135.00 ± 9.00 Aa	116.50 ± 13.80 Ab	87.12 ± 3.90 Ac	143.80 ± 4.68 Aa
Stem diameter (mm)					
_SL_Entisol	7.51 ± 0.61 Bd	17.75 ± 1.46 Ab	13.70 ± 2.53 Bc	8.79 ± 1.12 Bd	24.32 ± 3.29 Aa
_LS_Entisol	7.29 ± 0.58 Bc	17.43 ± 1.73 Ab	17.16 ± 2.04 Ab	9.74 ± 1.19 Bc	21.65 ± 2.31 Aa
_C_Oxisol_1_	7.13 ± 0.90 Bc	15.16 ± 1.19 Bb	12.15 ± 1.41 Bb	8.21 ± 0.66 Bc	18.51 ± 1.39 Ba
_SCL_Oxisol	7.27 ± 0.78 Bd	17.86 ± 3.45 Ab	13.32 ± 1.17 Bc	7.29 ± 0.98 Bd	24.57 ± 1.25 Aa
_C_Oxisol_2_	12.90 ± 2.18 Ac	20.38 ± 0.46 Aa	17.77 ± 3.18 Ab	13.84 ± 1.09 Ac	21.07 ± 0.58 Aa
Shoot dry weight (g pot^−1^)					
_SL_Entisol	4.75 ± 1.46 Bd	58.47 ± 9.78 Bb	22.07 ± 10.44 Bc	5.51 ± 1.17 Ad	119.41 ± 6.68 Aa
_LS_Entisol	4.12 ± 0.53 Bc	54.42 ± 3.00 Bb	47.48 ± 12.00 Ab	7.21 ± 1.98 Ac	109.79 ± 8.55 Aa
_C_Oxisol_1_	3.33 ± 1.03 Bc	39.67 ± 7.91 Cb	20.88 ± 4.22 Bb	5.35 ± 0.92 Ac	55.08 ± 8.38 Ca
_SCL_Oxisol	3.97 ± 0.25 Bd	51.10 ± 15.68 Bb	16.67 ± 3.70 Bc	3.97 ± 1.31 Bd	125.61 ± 15.61 Aa
_C_Oxisol_2_	12.96 ± 2.57 Ae	79.48 ± 11.67 Ab	46.70 ± 13.69Ac	21.13 ± 1.81Ad	99.70 ± 5.23Ba

Different lowercase letters within rows differ by Tukey’s test at α = 0.05, and different capital letters within columns differ by the same post-hoc test. Numbers represent the mean values (*n* = 4) followed by the standard deviation (SD).

_SL_*Entisol* sandy loam Entisol, _LS_*Entisol* loamy sand Entisol. _C_*Oxisol*_1_  clayey Oxisol, _SCL_*Oxisol* sandy clay loam Oxisol, _C_*Oxisol*_2_ clayey Oxisol.

*PP1* precipitated P,* PP2* precipitated P,* RP* reactive phosphate rock,* TSP* triple superphosphate.

### Phosphorus in soil solution

**Figure 2:**
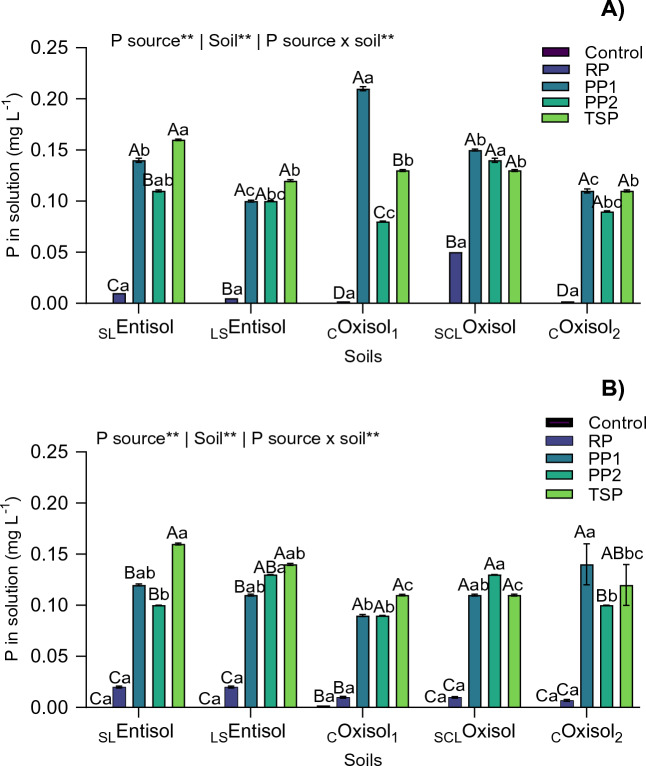
Phosphorus concentration in soil solution (soluble P) at 15 days (**A**) and 50 days (**B**) after fertilizers application at 120 mg P dm^−3^ rate in the form of alternative P sources (PP1 and PP2), reactive phosphate rock (RP), and triple superphosphate (TSP). Different lowercase letters within the same treatment (P source) differ by Tukey’s test at α = 0.05, and different capital letters within the same soil differ by the same post-hoc test. Numbers represent the mean values (*n* = 4) followed by the standard deviation (SD). ^**^p < .01. _SL_*Entisol* sandy loam Entisol, _LS_*Entisol* loamy sand Entisol. _C_*Oxisol*_1_  clayey Oxisol, _SCL_*Oxisol* sandy clay loam Oxisol, _C_*Oxisol*_2_ clayey Oxisol.

A pronounced difference was found for soil solution P among the tropical soils when P sources were applied. Initially, at 15 DAE, the highest values were found in the _C_Oxisol_1_ when PP1 source was applied (Fig. 2A). In this soil, the soluble P was 61.90%, 99.04%, and 38.09% higher than PP2, RP and TSP, respectively. After 30 days, TSP presented greater values in _SL_Entisol, _LS_Entisol and _CO_xisol_1_. Also, those alternative sources (PP1 and PP2) showed the highest soluble P values in the _SCL_Oxisol and _C_Oxisol_2_ (Fig. 2B).

### Nutrients accumulation in the shoot

The two-way ANOVA showed significant differences among the P sources for the nutrients’ accumulation in the shoots (Table 4). The highest values for P, N, S, Ca, and Mg were observed where TSP was applied, to all studied soils. However, when evaluating only alternative P sources, the highest values for P, N, and S accumulation were greater in the PP1, showing an increase of up to 37.05% and 75.98% (P), 34.39% and 72.07% (N); and 41.94% and 72.69% (S) in comparison to PP2 and RP, respectively. Among the studied soils, our results showed that maize plants cultivated in the _SL_Entisol, _LS_Entisol, and _C_Oxisol_2_ had the highest accumulated P in the shoots. For Ca and Mg, the highest values were found in the _SCL_Oxisol when PP2 was applied ‒ 44.31% and 69.48% for Ca and 46.23 and 75.79% for Mg, respectively, in comparison to PP1 and RP. It was not observed a significant response for K accumulation. When the soil types were compared, the highest values of accumulated K were found in the _C_Oxisol_2_. On the other hand, when comparing the P sources, the highest values were found for TSP followed by PP1 (Table 4).

**Table 4:** Nutrients accumulation (mean ± SD) in shoot biomass of maize crops cultivated in five soils, after application of alternative P sources.

Soils			P sources		
	Control	PP1	PP2	RP	TSP
P accumulation (mg plant pot^−1^)					
_ SL_Entisol	6.21 ± 2.45 Ad	154.91 ± 26.29 ABb	58.75 ± 28.97 Bc	8.94 ± 2.53 Cd	231.38 ± 23.90 Aa
_ LS_Entisol	5.39 ± 1.09 Ac	141.19 ± 14.88 BCb	113.15 ± 19.70 Ab	12.63 ± 4.15 ABc	214. 81 ± 14.83 Aa
_ C_Oxisol_1_	4.65 ± 1.96 Ac	90.14 ± 14.62 Db	49.33 ± 13.91 Bb	8.98 ± 0.98 Cc	133.70 ± 17.37 Ba
_ SCL_Oxisol	5.33 ± 0.44 Aa	112.72 ± 26.42 CDb	38.93 ± 7.81 Bc	5.65 ± 2.38 Cc	225.26 ± 31.82 Aa
_ C_Oxisol_2_	19.57 ± 11.98 Ac	186.93 ± 17.73 Aa	117.66 ± 25.32 Ab	44.99 ± 2.73 Ac	201.42 ± 26.90 Aa
N accumulation (mg plant pot^−1^)					
_ SL_Entisol	147.35 ± 8.02 Ad	1788.80 ± 26.26 ABb	695.55 ± 34.40 Bc	162.60 ± 3.41 ABd	2697.25 ± 33.78 ABa
_ LS_Entisol	116.17 ± 3.89 Ac	1657.67 ± 13.93 Bb	1420.52 ± 30.64 Ab	197.55 ± 4.84 ABc	2479.92 ± 32.25 Ba
_ C_Oxisol_1_	83.17 ± 5.79 Ac	988.10 ± 26.78 Cb	624.35 ± 11.06 Bb	136.45 ± 4.64 Bc	1582.15 ± 29.98 Ca
_ SCL_Oxisol	120.90 ± 3.27 Ac	1556.62 ± 49.42 Bb	503.52 ± 87.80 Bc	114.05 ± 6.52 Bc	3111.30 ± 32.22 Aa
_ C_Oxisol_2_	284.85 ± 14.7 Ad	2149.80 ± 15.23 Ab	1410.32 ± 29.80 Ac	600.42 ± 3.83 Ad	2703.92 ± 28.16 ABa
S accumulation (mg plant pot^−1^)					
_ SL_Entisol	15.12 ± 8.96 Ac	161.12 ± 27.92 Ab	55.90 ± 33.25ABCc	12.35 ± 5.25 Ac	374.12 ± 79.16 Aa
_ LS_Entisol	12.82 ± 4.48 Ac	117.85 ± 13.95 ABb	103.10 ± 29.69 Ab	14.80 ± 4.90 Ac	267.35 ± 42.30 Ba
_ C_Oxisol_1_	7.72 ± 7.51 Ac	67.70 ± 17.31 Bab	38.77 ± 9.78 BCbc	10.52 ± 5.99 Ac	105.20 ± 21.74 Da
_ SCL_Oxisol	12.25 ± 7.33 Ac	136.05 ± 66.05 Ab	34.22 ± 9.42 Cc	9.92 ± 6.80 Ac	261.22 ± 35.71 Ba
_ C_Oxisol_2_	21.05 ± 14.60 Ac	161.30 ± 18.04 Aa	93.65 ± 27.21 ABb	44.05 ± 5.60 Abc	200.65 ± 21.62 Ca
Ca accumulation (mg plant pot^−1^)					
_ SL_Entisol	34.52 ± 7.51 Ac	103.35 ± 35.07 ABCc	255.50 ± 65.34 ABb	36.87 ± 11.44 Ac	573.82 ± 122.45 ABa
_ LS_Entisol	28.72 ± 6.66 Ac	210.80 ± 77.80 Ab	243.10 ± 32.64 Bb	48.27 ± 14.59 Ac	419.47 ± 120.55 Ca
_ C_Oxisol_1_	20.47 ± 7.23 Ac	87.62 ± 19.64 BCbc	180.02 ± 96.59 Bab	30.57 ± 4.95 Ac	231.62 ± 63.13 Da
_ SCL_Oxisol	23.35 ± 3.67 Ac	80.22 ± 27.09 Cc	270.40 ± 71.66 ABb	27.12 ± 7.32 Ac	612.67 ± 105.79 Aa
_ C_Oxisol_2_	57.32 ± 29.39 Ac	202.37 ± 58.85 ABb	363.55 ± 70.13 Aa	111.02 ± 7.23 Bbc	463.75 ± 40.73 BCa
Mg accumulation (mg plant pot^−1^)					
_ SL_Entisol	28.35 ± 18.82 Ac	87.52 ± 33.97 Bc	202.52 ± 60.93 Bb	27.07 ± 12.65 Ac	502.75 ± 67.78 Ba
_ LS_Entisol	23.85 ± 15.69 Ac	156.82 ± 47.84 ABb	184.60 ± 22.66 Bb	34.97 ± 8.41 Ac	345.27 ± 83.42 Ca
_ C_Oxisol_1_	24.37 ± 19.47 Ac	113.82 ± 11.53 Bbc	164.07 ± 54.33 Bab	32.90 ± 16.64 Ac	245.75 ± 44.09 Ca
_ SCL_Oxisol	26.27 ± 19.38 Ac	90.52 ± 33.15 Bc	269.70 ± 75.15 Bb	30.92 ± 23.35 Ac	732.60 ± 96.79 Aa
_ C_Oxisol_2_	24.20 ± 30.91Ac	226.80 ± 82.04 Ab	421.87 ± 140.41 Aa	102.10 ± 18.02 Ac	481.60 ± 50.81 Ba
K accumulation (mg plant pot^−1^)					
_ SL_Entisol	1005.03 ± 758.32 b		Control	224.59 ± 129.63 c	
_ LS_Entisol	1352.53 ± 1010.41 a		PP1	1834.51 ± 418.73 a	
_ C_Oxisol_1_	861.36 ± 644.82 b		PP2	1216.60 ± 533.06 b	
_ SCL_Oxisol	986.66 ± 804.87 b		RP	369.85 ± 259.54 c	
_ C_Oxisol_2_	1598.80 ± 1056.88 a		TSP	2140.84 ± 714.09 a	

Different lowercase letters within rows differ by Tukey’s test at α = 0.05, and different capital letters within columns differ by the same post-hoc test. Numbers represent the mean values (*n* = 4) followed by the standard deviation (SD).

_SL_*Entisol* sandy loam Entisol, _LS_*Entisol*  loamy sand Entisol. _C_*Oxisol*_1_ clayey Oxisol, _SCL_*Oxisol*  sandy clay loam Oxisol, _C_*Oxisol*_2_ clayey Oxisol.

*PP1* precipitated P,* PP2* precipitated P,* RP* reactive phosphate rock,* TSP* triple superphosphate.

### Relative agronomic efficiency

**Figure 3:**
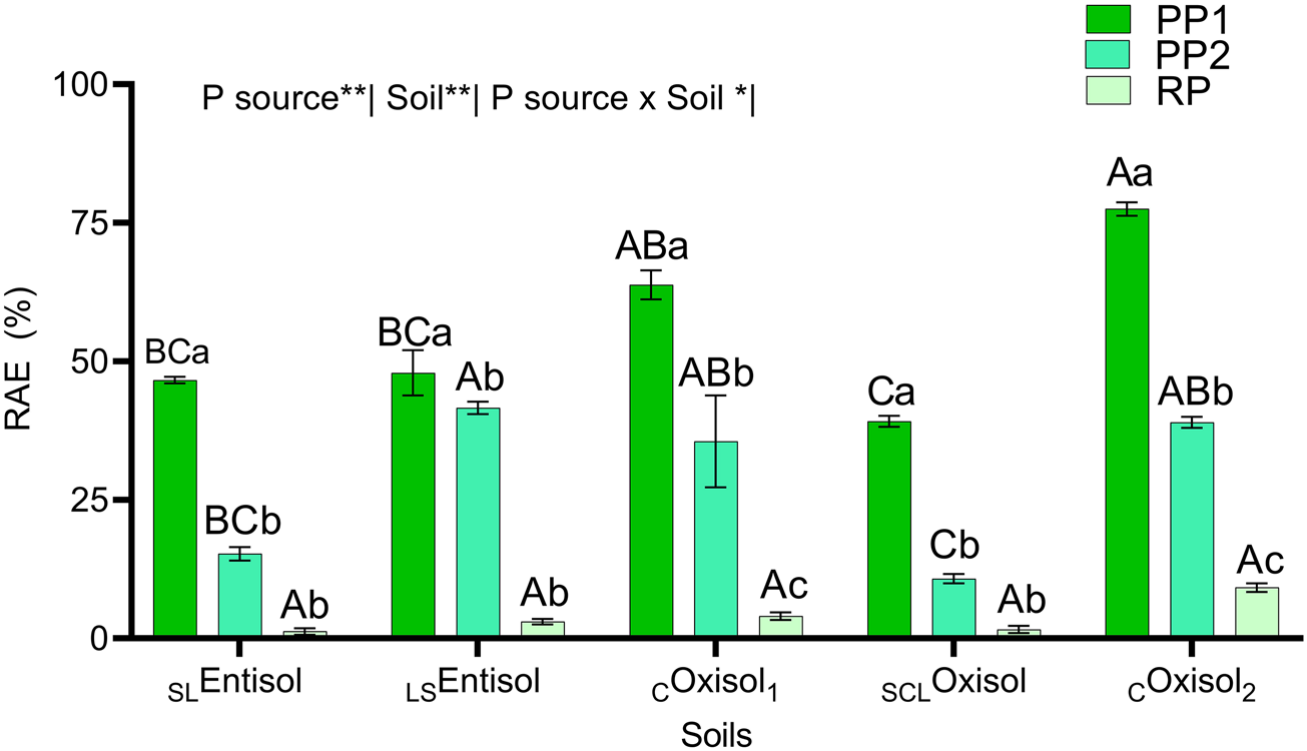
Relative agronomic efficiency of three alternative P fertilizers applied in five tropical soils, Botucatu, São Paulo, Brazil. ^*^p < .05. ^**^p < .01. _SL_*Entisol* sandy loam Entisol, _LS_*Entisol* loamy sand Entisol. _C_*Oxisol*_1_  clayey Oxisol, _SCL_*Oxisol* sandy clay loam Oxisol, _C_*Oxisol*_2_ clayey Oxisol.* PP1* precipitated P,* PP2* precipitated P,* RP* reactive phosphate rock.

Relative agronomic efficiency (RAE) of alternative P sources is shown in Fig. 3. The highest values were found in the _C_Oxisol_2_ when PP1 was applied. This source showed an increase of 49.75% and 88.14% in comparison to PP2 and RP.

### Multivariate analysis

**Figure 4:**
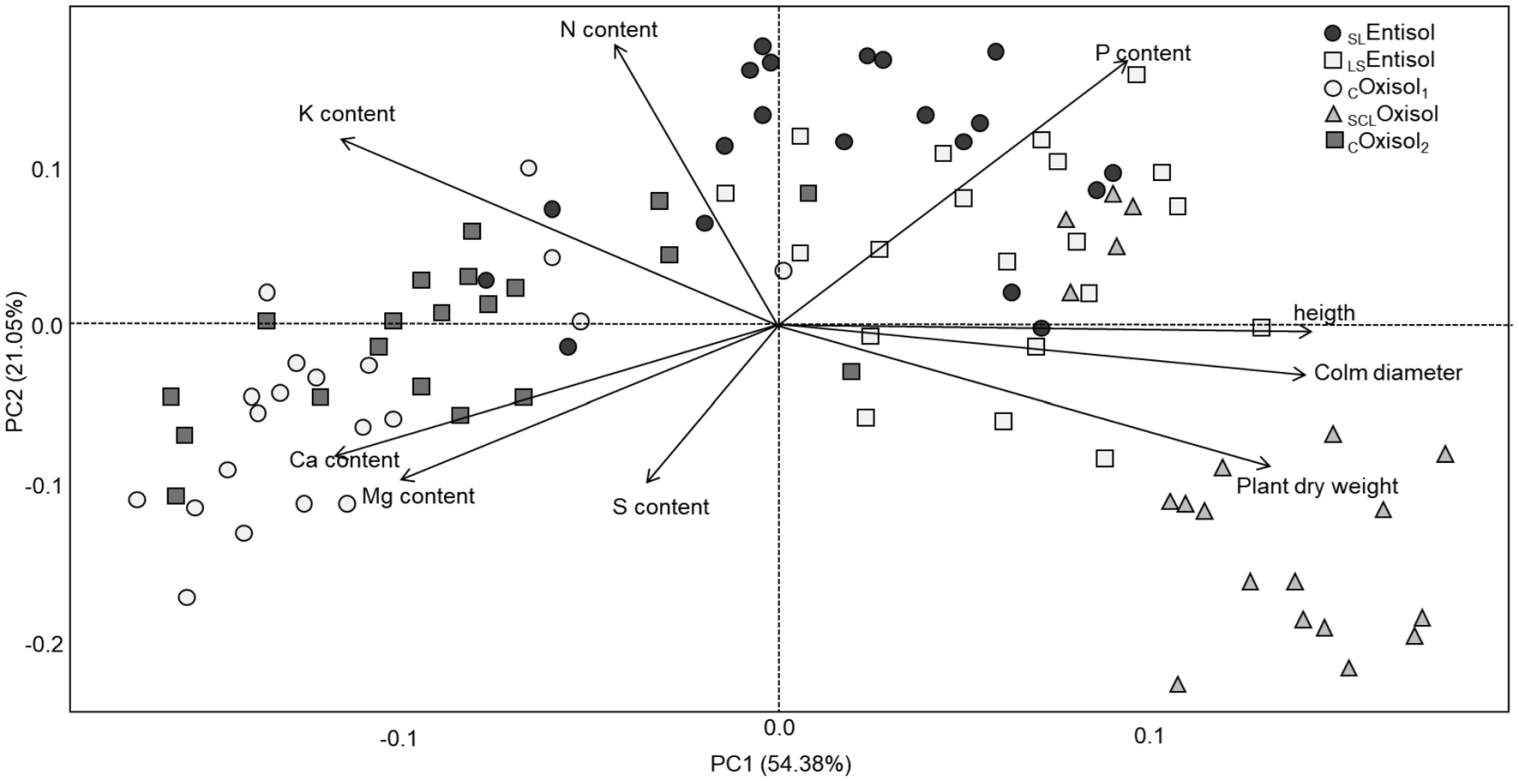
PCA score plot of plant attributes and plant nutrient contents in different tropical soils. Geometric figures represent samples from each plot for each studied soil. The two axes explained 72.43% (PC1 = 54.38% and PC2 = 21.05%) of the total variance. _SL_*Entisol* sandy loam Entisol, _LS_*Entisol* loamy sand Entisol. _C_*Oxisol*_1_ clayey Oxisol, _SCL_*Oxisol* sandy clay loam Oxisol, _C_*Oxisol*_2_ clayey Oxisol.

The principal component analysis (PCA) showed that K, N, P, Ca, Mg, S accumulated in plant tissues, height, stem diameter, and shoot dry weight were the main factors contributing to the variance of the samples (Fig. 4). The first two principal components (PC1 and PC2) accounting for 75.43% of the data variance were assumed as the most principal components. The analysis showed the following aspects: (i) a positive correlation between N and K accumulation; (ii) positive correlation between Ca and Mg accumulation; (iii) positive correlation between stem diameter and shoot dry weight (Fig. 4).

## Discussion

The results highlighted the effects of alternative P sources on maize crop nutrition and shoot biomass production in various Brazilian tropical soils. We aimed to understand how these sources enhance nutrient accumulation and morphological attributes in maize crops through a greenhouse experiment. The findings indicated that alternative P sources from manufactured fertilizers can increase soil P levels and nutrient uptake by maize plants in soils with varying clay content. Our results revealed significant differences among the studied P sources in terms of plant attributes (e.g., height, stem diameter, and shoot dry weight) and nutrient accumulation in plant tissues as well.

Several studies have emphasized the role of alternative P sources as a sustainable approach, primarily due to their slow release and positive impact on crop growth^28–30^. The increase in shoot dry weight is directly linked to P availability for crops associated with improved photosynthetic activity, including Rubisco activity and regeneration. On the other hand, low shoot dry weight can be attributed to a reduction in the maximum quantum yield related to the photochemical system, which occurs under conditions of limited P availability^31^.

The P use efficiency (PUE) in tropical soils is very low and it is mainly linked to soil chemical properties such as PMAC, clay content, and soil pH. These factors play a crucial role in determining P availability because they are closely associated with the adsorption process^6^. In our study, we observed that the PP1 (precipitated phosphate 1) and PP2 (precipitated phosphate 2) sources exhibited slightly lower plant morphological and nutritional attributes, and agronomic efficiency when compared to conventional sources like TSP. This finding supports our hypothesis that alternative sources can enhance P availability for crops, potentially reducing reliance on TSP application in tropical conditions. Notably, our results indicated that even when PMAC ranged from 113.51 to 1015.4 mg kg^−1^, the alternative sources performed similarly to TSP. This similarity can be attributed to the slow release of these alternative sources, which may result in increased P availability for crops during periods of high demand^32^.

Some studies have described that alternative phosphorus sources (e.g., precipitated phosphate) could exhibit a residual effect. For instance, studies by Leal et al.^10^, evaluating alternative phosphorus sources from the manufacturing of TSP, showed an increase in biomass production of grasses under acidic conditions, and after successive cuts, the residual effect showed greater values when compared to TSP. Results obtained by Bogdan et al.^33^ showed that alternative phosphorus sources can gradually release phosphorus for up to seven months, demonstrating higher availability compared to TSP by up to 42% during the growth of perennial ryegrass. Ashekuzzaman et al.^34^ demonstrated that the application of precipitated phosphorus sources derived from industrial processes can exhibit a residual effect and increase phosphorus bioavailability by up to 109%.

The stability of the soil solution during the sampling period (e.g., 15 and 30 days) in a clayey soil (_C_Oxisol_2_) is linked to soil physical–chemical properties, including soil organic matter (SOM), PMAC, clay content, and soil pH. According to Wang et al.^35^, the rise in soil pH (e.g., 5.5–6.5) critically influences phosphorus (P) pools in soils. This factor regulates the solubility and distribution of minerals, altering adsorption dynamics in clayey soils. In studies by Sandim et al. ^14^, the PP1 source increased the moderately labile P pools, extracted with HCl and formed by Ca–P. Calcium (Ca) presence may result from lime application or be inherent in the source composition.

Studies by Wang et al.^35^ demonstrated that root activity governs phosphorus solubility, availability, and pools, influencing soil microbial activity through root exudation. Finally, root activity (e.g., organic acid exudation) can chelate Ca present in calcium-phosphate bonds (e.g., through hydroxyl and carboxyl groups) or compete with phosphate for adsorption sites, thereby increasing phosphorus solubility^36^. According to Veloso et al.^37^, in clayey Oxisol, the modest stress (e.g., low phosphorus release at the time) can increase enzymatic activity (e.g., acid phosphatase) by plants, microbial activity, or interactions between them. Thus, another possible explanation for greater values of PP1 and PP2 in _C_Oxisol_2_ can be a soil organic matter content supporting microbial activity and increasing enzymatic activity or the positive feedback between plant and soil biota, increasing P release of alternative P sources.

This finding helps explain the greater biomass production observed when using this source (Table 3), as this P fraction remains in equilibrium with the soil solution, making it readily available to crops during their development. Several studies^38–40^ have shown that alternative P sources can enhance plant growth and biomass production. This effect can be attributed to changes in the plant-soil continuum, influencing P dynamics in the plant rhizosphere through three main mechanisms: (i) Plants can modify their root environment by extruding H^+^ ions, leading to rhizosphere acidification, which enhances the solubilization of P from these sources^41^; (ii) Root exudates stimulate microbial activity, including enzymes like phytase and acid phosphatase, especially in low P conditions; (iii) The symbiosis association with arbuscular mycorrhizal fungi (AMF), increasing plant growth and improving soil structure and nutrient contents (mainly P) thus increasing plant productivity^42^.

However, as described by Ai et al.^43^, root exudates may promote microbial metabolism without increasing microbial biomass P utilization. This implies that there may not be competition between microorganisms and plant roots for P in P-poor soils or when slow-release P sources are applied. Furthermore, Rezakhani et al. ^44^ demonstrated that low-solubility P fertilizers can boost shoot dry weight by stimulating solubilizing microorganisms, which not only enhance P availability but also influence the uptake of other elements like silicon (Si). This, in turn, promotes increased shoot and root biomass, creating a positive feedback loop in the rhizosphere.

The P application resulted in increased nutrient accumulation, particularly when alternative P sources were used, leading to higher levels of N, S, and Mg in plant tissues when compared to TSP. It is well-established that P content in plants is closely associated with the uptake and accumulation of other nutrients^45^. In fact, P supplementation has been shown to enhance nutrient content in maize crops ^3^. Alternative P sources have the capacity to release nutrients that are part of their composition, often facilitated by microbial solubilization. Additionally, P plays a vital role in plant physiology by contributing to the ATP pools, providing the energy needed for nutrient uptake and biomass production. The application of P fertilizers can also expose plant roots to a larger surface area, thereby increasing nutrient absorption. Consequently, the observed increases in key nutrients such as N and Mg are directly linked to photosynthetic processes and overall biomass production^46^.

The nutrient accumulation was most pronounced when maize crops were cultivated in soils characterized by low and mean PMAC levels. Recent reports have described that higher clay contents may contribute to SOM stabilization in soils^47–49^. Recently, Liu et al.^49^, found that hydroxy-interlayered clay minerals, creating a hydroxy-Al polymer with organic particles, reduced the oxidation. Here, we found that _C_Oxisol_2_ showed a similar clay content when compared to _C_Oxisol_1_, however, with much greater SOM content (Table 1). This observation suggests that the buffer capacity of tropical soils may influence the nutrient accumulation. Specifically, sandy and medium texture soils and clayey soils with high SOM (_SL_Entisol, _SCL_Oxisol, and _C_Oxisol_2_) sources led to increases in both nutrient content and shoot dry weight production (as showed in PCA). In soils with higher PMAC values (_C_Oxisol_1_), the P buffer capacity may become a limiting factor for production. According to Yang et al.^50^, the SOM dynamics can regulate P adsorption/desorption and control its availability under certain conditions. Notably, crops with rapid growth cycles like maize tend to be more productive in loam and sandy soils than in clayey soils^51^. Therefore, optimizing PUE under these conditions is paramount, with considerations for slow-release fertilizers and alternative P sources.

The PCA provided valuable insights into the influence of soil texture on nutrient accumulation and shoot dry weight production under tropical conditions, elucidating the intricate relationship between soil type and key plant parameters, including nutrient uptake. We observed that soils with a moderate clay content may exhibit a harmonious equilibrium in their charge properties, thereby facilitating heightened nutrient accumulation in plant shoots, particularly P, K, and N. Conversely, sandy soils (_SL_Entisol) showed similar correlations with plant attributes, possibly due to their lower adsorption capacity (lower surface charges than clayey soils) and an increased concentration of nutrients in the soil solution without losses via leaching (Fig. 4). This resulted in a significant accumulation of these nutrients. These findings agree with studies that report the importance of accounting for soil texture when considering P fertilization, plant attributes, and nutrient accumulation strategies^52^.

Our greenhouse experiment showed which alternative P source had potential to enhance plant height, shoot dry weight, stem diameter, and nutrient accumulation. As far as we know, this is the first report on the agronomic efficiency of by-products from phosphate fertilizer production in Brazilian tropical soils. These results could be of great importance for the valorization of these by-products in agriculture, reducing costs associated with commercial sources, increasing productivity, and reducing Brazil's international dependence on phosphate fertilizers. Finally, further studies should be conducted to understand how the application of alternative P sources can influence the rhizosphere microbiome and increase P availability.

## Conclusion

The precipitated phosphate sources (PP1 and PP2) exhibited similar behavior to the commercial source TSP, suggesting their potential for reducing reliance on TSP and enhancing soil fertility in tropical conditions (_SCL_Oxisol, _C_Oxisol_2_, and _SL_Entisol). The addition of supplemental phosphorus (P) can boost nutrient accumulation in maize crops, irrespective of the source used, although they may influence crop responses differently. Consequently, it is imperative to refine P management strategies tailored to specific sources and soil textures in tropical regions. Moreover, conducting further research at the field scale is essential to validate these findings in real-world scenarios, accounting for variables such as weather and landscape variations. Comprehensive studies under controlled conditions are also necessary to deepen our understanding of the soil microbiome's role in P dynamics within tropical soils following the application of alternative P sources, like those examined in this study.

## Data Availability

The data that support the findings of this study are available from the corresponding author upon reasonable request.
